# Larval Exposure to the Juvenile Hormone Analog Pyriproxyfen Disrupts Acceptance of and Social Behavior Performance in Adult Honeybees

**DOI:** 10.1371/journal.pone.0132985

**Published:** 2015-07-14

**Authors:** Julie Fourrier, Matthieu Deschamps, Léa Droin, Cédric Alaux, Dominique Fortini, Dominique Beslay, Yves Le Conte, James Devillers, Pierrick Aupinel, Axel Decourtye

**Affiliations:** 1 ACTA, ICB-VETAGROSUP, 1 avenue C. Bourgelat, 69280, Marcy l’Etoile, France; 2 INRA, UR 406 Abeilles et Environnement, 228 Route de l'Aérodrome, CS 40509, 84914, Avignon, Cedex 9, France; 3 UMT PrADE, CS 40509, Avignon, France; 4 INRA, UE1255, UE Entomologie, Station du Magneraud, CS 40052, 17700, Surgères, France; 5 CTIS, 3 chemin de la gravière, 69140, Rillieux La Pape, France; 6 ACTA, 228 Route de l'Aérodrome, CS 40509, 84914, Avignon, Cedex 9, France; 7 ITSAP-Institut de l’abeille, 228 Route de l'Aérodrome, CS 40509, Avignon, France; University of North Carolina, Greensboro, UNITED STATES

## Abstract

**Background:**

Juvenile hormone (JH) plays an important role in honeybee development and the regulation of age-related division of labor. However, honeybees can be exposed to insect growth regulators (IGRs), such as JH analogs developed for insect pest and vector control. Although their side effects as endocrine disruptors on honeybee larval or adult stages have been studied, little is known about the subsequent effects on adults of a sublethal larval exposure. We therefore studied the impact of the JH analog pyriproxyfen on larvae and resulting adults within a colony under semi-field conditions by combining recent laboratory larval tests with chemical analysis and behavioral observations. Oral and chronic larval exposure at cumulative doses of 23 or 57 ng per larva were tested.

**Results:**

Pyriproxyfen-treated bees emerged earlier than control bees and the highest dose led to a significant rate of malformed adults (atrophied wings). Young pyriproxyfen-treated bees were more frequently rejected by nestmates from the colony, inducing a shorter life span. This could be linked to differences in cuticular hydrocarbon (CHC) profiles between control and pyriproxyfen-treated bees. Finally, pyriproxyfen-treated bees exhibited fewer social behaviors (ventilation, brood care, contacts with nestmates or food stocks) than control bees.

**Conclusion:**

Larval exposure to sublethal doses of pyriproxyfen affected several life history traits of the honeybees. Our results especially showed changes in social integration (acceptance by nestmates and social behaviors performance) that could potentially affect population growth and balance of the colony.

## Introduction

In holometabolous insects like honeybees, hormonal activity regulates important processes such as development, reproduction but also age-related division of labor (polyethism) between individuals [1,2]. In the typical pattern of age-related division of labor, young bees first perform inside-nest activities such as brood rearing, cell cleaning and queen care, and as they age, they switch to outside nest activities such as guarding and foraging [3,4]. Worker age-polyethism is however flexible and individuals can accelerate, delay or reverse their behavioral development according to changing environmental or social conditions [5].

Juvenile hormone (JH) plays an important role in the regulation of this temporal polyethism [5–8]: JH titer increases with behavioral development and is higher in foragers than in nurse bees [5,9]. The plasticity in behavioral development is also associated with JH titer changes [5,10].

In their natural environment, honeybees can be exposed to JH analog chemicals. These endocrine disruptors have been developed for insect pest and vector control and belong to the insect growth regulator (IGR) category. JH analogs affect development of target insects by disrupting the molting process, which results in damage or death [11]. They are therefore considered to be of higher risk for larvae than for adults in non-target insects (for a review see [11]). For example, in honeybees, several studies have shown that larval exposure to JH analogs can result in death or larval ejection by workers, or in malformed larvae and resulting adults [11–13]. In Europe, the official regulatory process requires risk evaluation of IGR compounds like JH analogs on honeybee brood [14]. But JH analogs have also been reported to have effects on adult bees. For example, some JH analogs used to study the role of JH in the social organization of adult bees, showed effects on the timing and frequency of social tasks occurrence like brood and queen care, food storage, nest maintenance and the onset of foraging [15,16]. However, while JH is cited as a main driver mechanism of polyethism in honeybees [5], there is nearly no information about the effects of a chronic larval exposure to sublethal doses of a JH analog insecticide on adult life history traits. The need to perform studies that can reliably detect behavioral effects in individuals exposed to a compound showing IGR activity during the larval stage has already been raised by Desneux et al. [17].

We therefore studied the effects of repeated larval exposure to a JH analog on the development and social behaviors of resulting adults. Repeated exposure was tested because it is closer to honeybee brood exposure conditions in the field [18,19]. We aimed to follow the effects of sublethal doses of pyriproxyfen, an IGR used in crop protection against pests (e.g. whiteflies, cochineal insects …) and in vector control against mosquito larvae. We used a methodological approach combining recent and standardized honeybee brood rearing technique in the laboratory [20–23] and a behavioral study on adults within a colony [24,25] placed in semi-field conditions. The impact of pyriproxyfen was therefore determined throughout the worker’s life cycle by analyzing larval development, behavioral traits and social acceptance by nestmates. Since acceptance is related to social recognition by nestmates notably via colony-specific cuticular hydrocarbon (CHC) profiles [26,27], we also analyzed the effects of pyriproxyfen on the production of such chemical profiles.

## Materials and Methods

### Honeybees

Experiments were conducted for 6 weeks on honeybees (*Apis mellifera* L.) originating from 4 colonies from the ACTA apiary (Marcy L’Etoile, France) (three for producing larvae and sampling and one for receiving emerging adults for observations). The colonies were reared in the spring from artificial swarms and headed by 1-year-old mated queens obtained from a professional beekeeper with an organic management strategy. All queens had the same mother and were mated at the same queen mating station. The colonies were in 10-comb Dadant hives (three brood combs, four honeycombs and three empty combs).

### Larval rearing procedure

**Fig 1:**
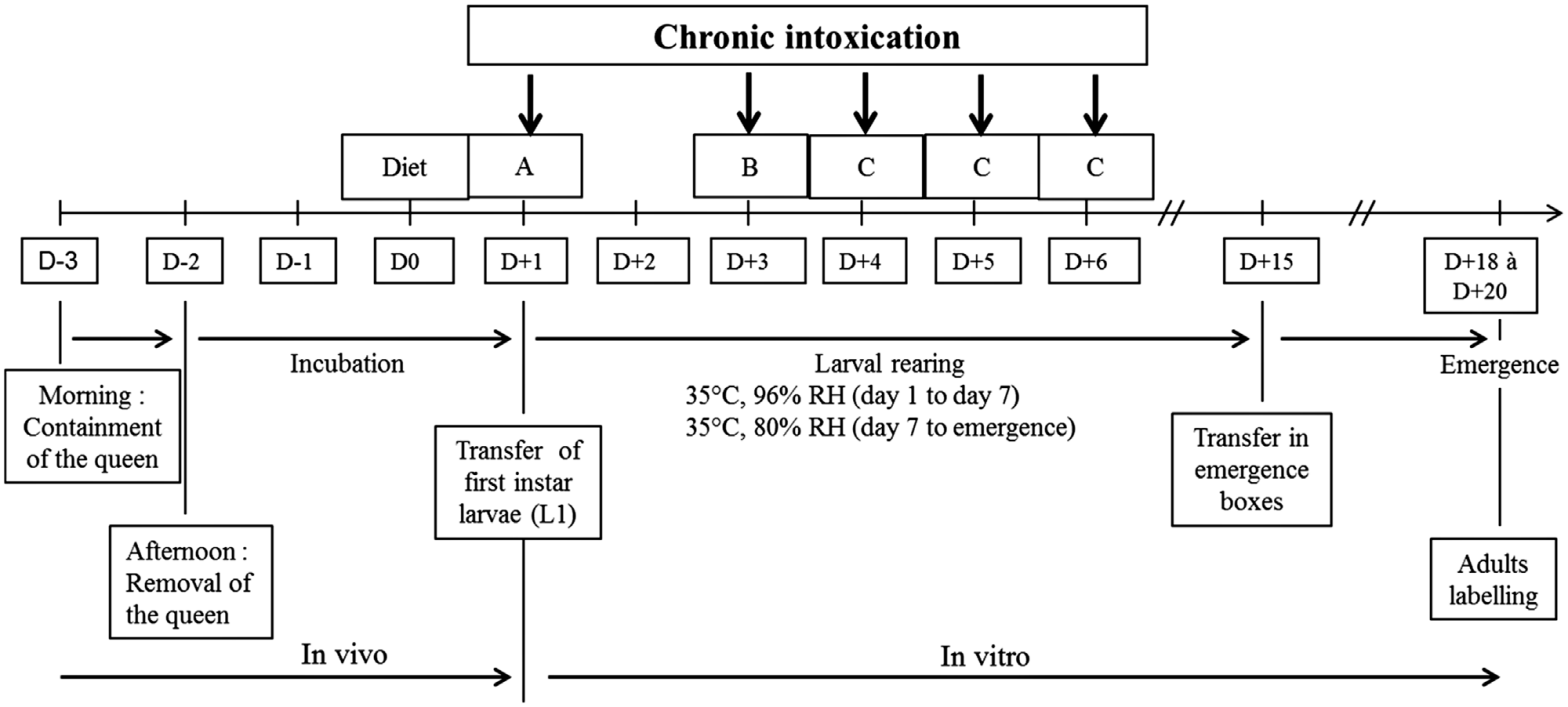
Larval rearing procedure.

We used *in vitro* larval rearing following the procedure of Aupinel et al. [20–22] (Fig 1). The queen was confined for 28 hours within the colony on an empty comb placed in an excluder cage (44.5 x 31.5 x 5.0 cm) to lay eggs. After removing the queen, the frame with its excluder device was returned to the middle of the brood chamber for three days. On the third day, the frame containing the first instar worker larvae (L1) was removed from the colony and brought to the laboratory at 25°C. Each larva was transferred to a plastic queen-starter-cell used in beekeeping for queen rearing and the cells were set in a 48-wells cellular culture plate. From day 1 to day 7 (pre-pupal stage) the larvae plates were kept in an incubator at 35°C and 96% RH. After day 7, the larvae were moved to a second incubator at 35°C and 80% RH. At day 15, before adult emergence, the plates were placed in plastic boxes fitted with feeders containing sugar syrup (50% w/w) and pollen.

Three diet compositions (A, B, C) were used for larval feeding, all containing 50% (w/w) royal jelly and 50% (w/w) sugar solution. In A, B and C, the sugar solution contained 24% sugar and 2% yeast extract, 30% sugar and 3% yeast extract, and 36% sugar and 4% yeast extract (w/w), respectively. The larvae were fed from day 1 (L1 stage) to day 6, except on day 2, with the three diet compositions (Fig 1): day 1 with diet A, day 3 with diet B and days 4 to 6 with diet C.

The experiment was conducted with larvae from 3 colonies. One treatment modality per plate and colony was tested giving a total of 3 plates per modality (144 larvae per treatment modality). Mortality was recorded from day 4 to day 7 for the larval stage, from day 8 to day 15 for the pupal stage and from day 18 to day 20 for the adult stage.

### Pyriproxyfen exposure

Technical-grade pyriproxyfen (98% pure) was purchased from Cluzeau Info Labo (France). Pyriproxyfen was tested at two different sublethal concentrations which were determined by preliminary experiments (data not shown). Stock solutions were prepared in acetone (99% pure, VWR) to a concentration of 0.067 g.L^-1^ and 0.0223g.L^-1^. Solutions were added to the larvae diet from day 1 to day 6 (Fig 1). Demineralized water and acetone controls were included. A water control aims to check the sanitary quality of the honeybees and to highlight possible acetone toxicity. The volumes of the control and treated solutions never exceeded 0.5% of the final diet volume. Each larva was fed a cumulative volume of 0.8 μl of the pyriproxyfen solution in food. Then, pyriproxyfen was tested at a cumulative dose of 54 ng and at a dose three times lower of 18 ng per larva, giving equivalent to concentrations in the diet of 305 ppb and 101 ppb, respectively. Finally, the two pyriproxyfen stock solutions prepared in acetone were analyzed (GIRPA, France) using the HPLC/MS technique (limit of quantification = 0.001 g L^-1^) to detect real concentrations. The concentrations of pyriproxyfen analyzed were 0.071 g. L^-1^ and 0.029 g. L^-1^ (actual concentrations in the diet of 321 and 129 ppb, respectively) giving actual cumulative doses of 57 and 23 ng per larva for the highest and lowest treatments, respectively.

### Adult emergence and reintroduction into a colony

Emerging workers were collected daily from day 18 to day 20 (Fig 1) and individually labeled with colored and numbered tags. A drop of gum arabic, drying within one minute, was used to glue the tag on the back side of the thorax in such a way that the wing movements were not hampered. Then the bees were placed in the incubator at 35°C and 50% RH for 24 hr before being reintroduced into a colony. Emerging bees with malformations (atrophied or deformed wings) were counted but were excluded from the groups used for investigating social behavior performance.

One-day-old tagged bees were then reintroduced from day 19 to day 21 in a four-frame glass-wall observation hive (2 frames high and 2 frames wide) containing a colony of about 6,000 honeybees and a fertile 1-year-old queen. This observation hive was enclosed in an outdoor flight cage (2.5 m × 2.5 m, 2 m high) covered with an insect-proof cloth (2 mm × 2 mm mesh). The ground was covered with a plastic sheet to collect dead bees.

The observation hive comprised 2 open and sealed brood combs, 1 honey and pollen comb and 1 empty comb for food storage. Food was delivered every day using an artificial feeder positioned about 1 m from the hive entrance. Before each observation, the feeder was placed and filled with 150 ml of sucrose solution (50% w/w) and 20 g of multi-floral pollen. The feeder was removed after each behavioral observation period.

### Rejection rate

After bee reintroduction, we recorded aggressive behaviors, i.e. rejection of tagged bees from the colony by nestmates (biting and pushing), for two hours per day (from 9:00 to 11:00 a.m.). These observations were recorded for five days after the first reintroduction. Since all tagged bees that were victims of aggressive behavior by nestmates were found dead on the ground during this period, we could establish a rejection rate per treatment group. In addition to the effect of pyriproxyfen treatment, other factors related to rearing in lab or the bees’ confinement in the flight cage might have increased the rejection phenomena.

### Behavioral and chemical analysis of adult bees

#### Cuticular hydrocarbon analysis

To determine whether pyriproxyfen could modify the cuticular hydrocarbons involved in social recognition and acceptance by nestmates, every day we collected newly dead tagged bees and stored them at -20°C. Hydrocarbons were extracted by individually immersing the bees for 5 minutes in a solution composed of 1,900 μL of isohexane and 100 μL of eicosane (C20) at 25 ng/μL as an internal standard. The details of the extraction and analysis with gas chromatography coupled with mass spectrometry are described in McDonnell et al. [28].

#### In-hive behavioral observations

To record bee behaviors, a grid with 5 x 5 cm squares was drawn onto the two glass walls of the hive. We used the “scan sampling” method to record behaviors [24]. This method consists of recording the behavior of the first tagged bee present in each square. Independent data are thus obtained. The behavior of each tagged bee observed in a square is recorded with an ethogram already described by Kolmes [24] and Matilla and Otis [25]. We quantified several distinct social behaviors: ventilation, brood care (physical contact with eggs, larvae or capped brood, indicating brood inspection or feeding), interactions with adult nestmates (antennal contact, feeding or cleaning), and contact with food stocks (food inspection or storage). Other observed behaviors like self-grooming, walking (in all directions at any speed and without contact) and inactivity (bee standing on the comb) were grouped according to Herbers and Cunningham [29] in “non-social behaviors”.

These behavioral observations were recorded daily for 15 days for one half-hour in the morning (within the 11:00 to 12:30a.m. time period) and one half-hour in the afternoon (within the 2:00 to 4:00 p.m. time period). Observations began one day after the last bee introduction. In summary, aggressive behaviors were recorded for 5 days starting after the first bee introduction, and social and non-social behaviors were recorded for 15 days starting 3 days after the first bee introduction.

### Statistical analysis

Data were analyzed using the statistical software R version 3.0.1 [30]. The effects of treatments on bee mortality during the larval, pupal and adult stages and on the number of bees rejected from the colony were assessed using a χ² test in a contingency table procedure (P < 0.05). To analyze the development time, a Kruskall-Wallis test (P < 0.05) was used. A Mann-Whitney U test with Bonferroni correction was then performed to assess pairwise differences between treatments.

Regarding adult behaviors, we performed two analyses: one including all control and treated bees during the first 5 days, and one including bees of all ages (overall observation period) for controls and 23 ng pyriproxyfen modalities since no further behavior was observed for bees exposed to 57 ng of pyriproxyfen five days after reintroduction. For both periods, we used a generalized linear mixed-effect model (GLMM) logistic regression to analyze the occurrence of social and non-social behavior using treatment and age as fixed factors. GLMM was performed with the binary (0 *vs* 1) social behavior data using a binomial distribution with canonical logit link function. The colony origin of the honeybees (3 colonies coded A, B or C) was considered as a random factor in the model. The model fitting procedure used the Laplace approximation to approximate likelihood. The significant effect of fixed explanatory variables as well as their interaction was determined from Wald tests (P < 0.05). Analyses were performed with the lme4 package for GLMM logistic regression [31].

For cuticular hydrocarbon analysis, only peaks that were reproducibly quantifiable in all samples were used for statistical analysis. The area of each peak was first standardized according to Reyment [32]. To determine whether treated and control bees could be distinguished on the basis of their cuticular profiles, we compared their chemical profile using a stepwise discriminant analysis (Statistica 8.0., StatSoft Inc.). The Wilks’λ value and the percentage of correct assignments were used to estimate the validity of the discriminant function. Pairwise-squared Mahalanobis distances between all groups were determined to estimate the chemical distances between each group. The effect of treatments on the relative proportion of each compound was determined using Kruskal-Wallis tests (P < 0.05).

## Results

### Mortality during development

During the larval stage, the cumulative mortality of water control bees was low (9.72%, Table 1) and below the standard acceptance threshold of 15% required to validate the *in vitro* larva-rearing assay [23]. Mortality during the other developmental stages was also low (Table 1) and cumulative mortality did not differ significantly between water or acetone control bees, or between the control groups and the pyriproxyfen-treated bees (Table 1; P > 0.05).

**Table 1:** Percentage of cumulative mortality during the larval, pupal, and adult stages in control and pyriproxyfen-treated bees.

	Control water	Control acetone	Pyriproxyfen 23 ng	Pyriproxyfen 57 ng	χ² tests	*P*
Larval stage (day 7)	9.72	9.72	11.11	11.11	0.30	0.96
Pupal stage (day 15)	14.58	18.75	15.97	14.58	1.24	0.74
Adult stage (day 20)	22.22	29.86	24.31	24.31	2.46	0.48

For each treatment group and developmental stage, percentages were established from a total of 144 larvae.

### Development time

**Fig 2:**
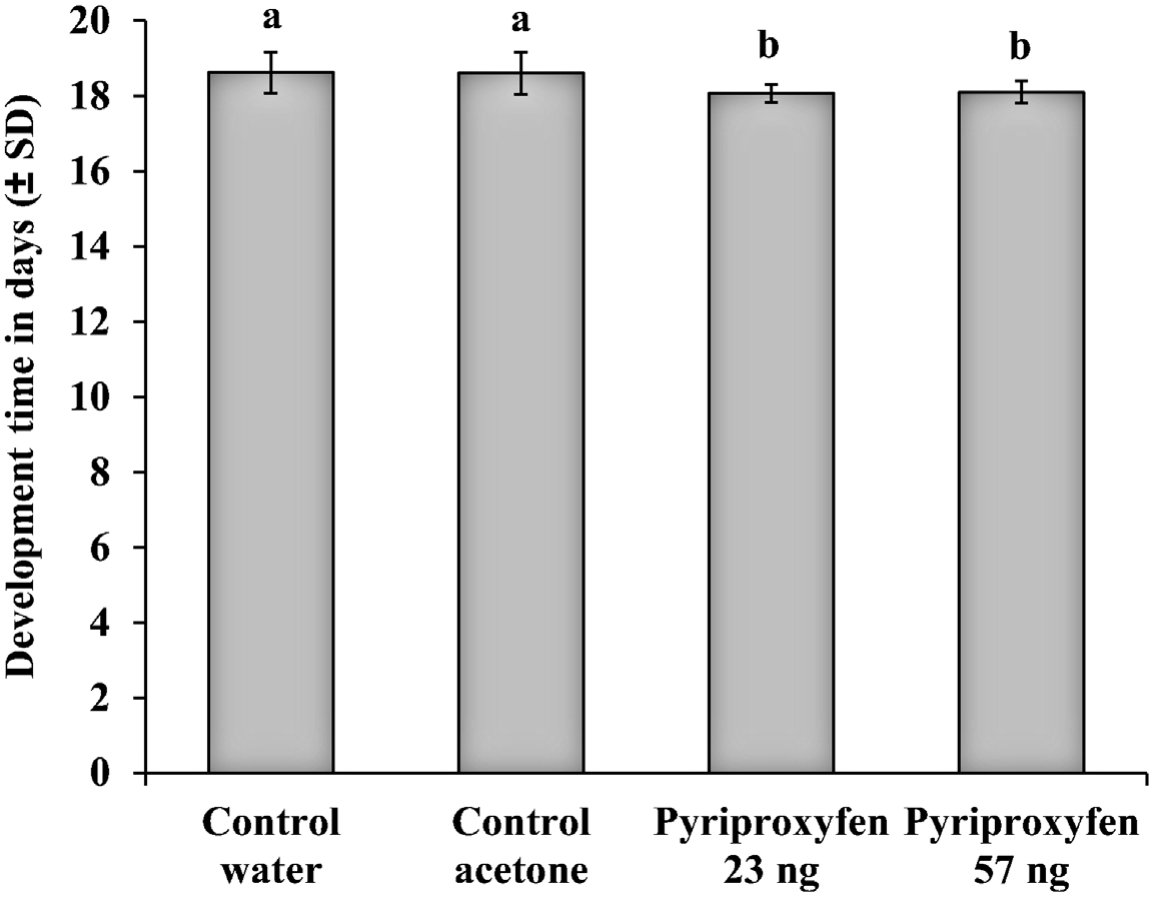
Development time of worker bees (L1 stage to adult emergence) according to treatments. Means ± SD are shown. Letters indicate significant differences (Mann-Whitney tests; P < 0.001).

We found a significant effect of treatments on the development time of bees (Kruskall-Wallis test, P < 0.001). Bees exposed to doses of 23 or 57 ng of pyriproxyfen had a slightly shorter development time than water or acetone control bees (Mann-Whitney tests, P < 0.001; Fig 2).

### Malformations

The proportion of bees exhibiting malformations (damaged or atrophied wings) did not differ between control groups (16.07% and 13.86% for control water (n = 112) and acetone (n = 101), respectively; χ² = 0.07, P = 0.80) but was significantly increased by the highest dose of pyriproxyfen (34.86% (n = 109); water vs pyriproxyfen 57 ng: χ² = 9.34, P < 0.01; acetone vs pyriproxyfen 57 ng: χ² = 11.31, P < 0.001). The rate of malformed bees did not differ between the highest dose and the lowest dose of pyriproxyfen (22.94% for pyriproxyfen 23 ng (n = 109); χ² = 3.22, P = 0.07) and between the lowest dose and the control groups (water vs pyriproxyfen 23 ng: χ² = 1.25, P = 0.26 and acetone vs pyriproxyfen 23 ng: χ² = 2.29, P = 0.13).

### Observations on adult honeybees

#### Rejection rate

The rejection rate of control bees was low and did not differ between water (6.38%, n = 94) and acetone exposed bees (8.05%, n = 87, χ² = 0.02, P = 0.89). These rejection rates could reflect the effect of experiments conditions (larval rearing in lab and bees’ confinement in the flight cage). The rejection rate increased with the exposure dose (38.10% and 74.65% for pyriproxyfen 23 ng (n = 84) and pyriproxyfen 57 ng (n = 71), respectively). Exposed honeybees were significantly more rejected when exposed to pyriproxyfen (water vs pyriproxyfen 23 ng: χ² = 24.71, P < 0.001; acetone vs pyriproxyfen 23 ng: χ² = 20.25, P < 0.001; water vs pyriproxyfen 57 ng: χ² = 79.11, P < 0.001; acetone vs pyriproxyfen 57 ng: χ² = 70.83, P < 0.001). Five days after the first reintroduction, we did not observe any more aggressive and rejection behaviors by nestmates. By this time, there were nearly no bees exposed to the highest dose in the observation hive.

#### Cuticular hydrocarbon analysis

**Fig 3:**
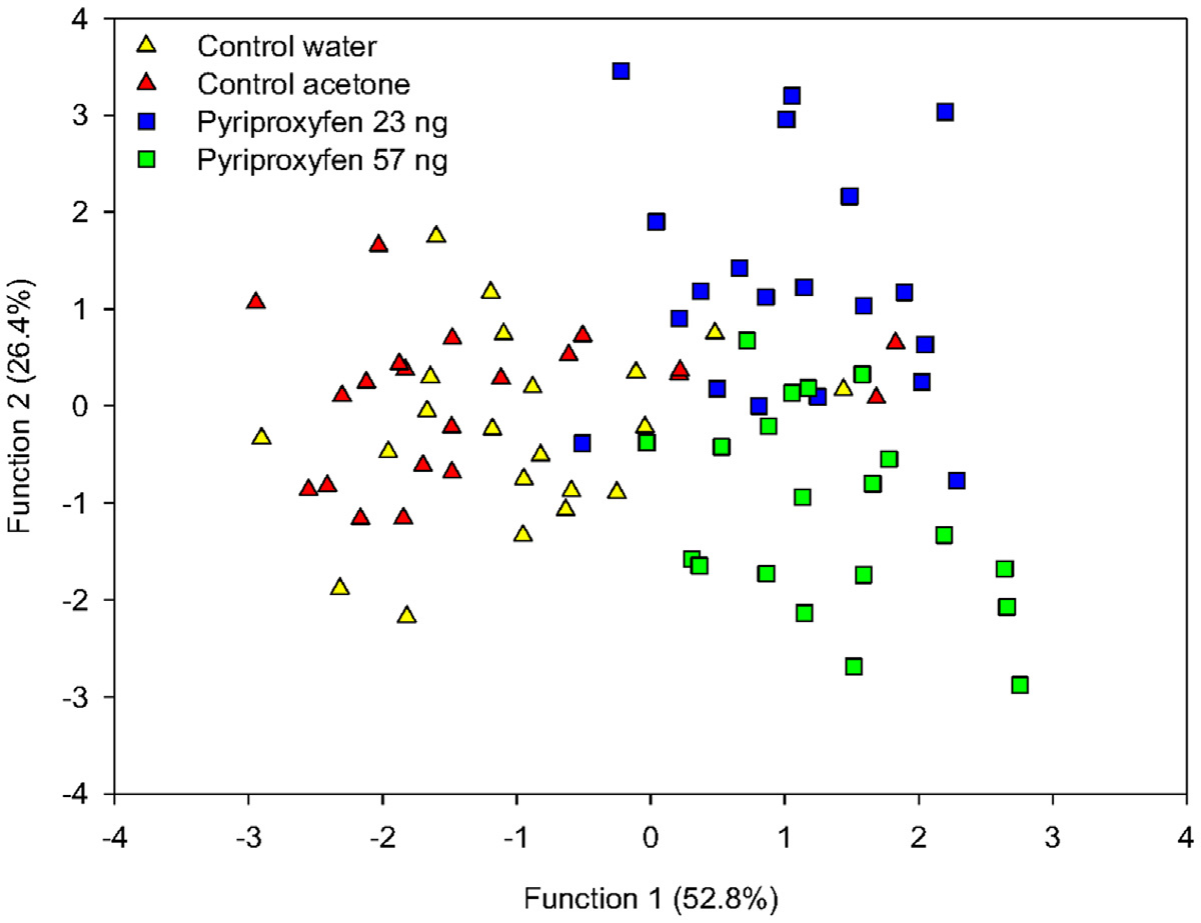
Cuticular hydrocarbon profiles of pyriproxyfen- and non-treated bees. Discriminant analysis was performed on 21 bees per control (water and acetone) and 20 bees per pyriproxyfen treatment (23 ng and 57 ng).

We did not find new compounds in the cuticular hydrocarbon profiles related to pyriproxyfen treatment compared to control groups (S1 Table). However, the discriminant analysis, revealed a highly significant effect of treatments on the CHC profiles of bees (Wilks’λ = 0.33, F_33,201_ = 2.8, P < 0.001; Fig 3) and it was possible to correctly assign 71.4 to 80% of the bees from the different groups based on their chemical profiles (Table 2). We further analyzed the effect of treatments by determining the pairwise-squared Mahalanobis distances between the CHC profiles of different treatment groups. The chemical profiles were neither different between control groups after Bonferroni's correction (P > 0.01) nor between bees treated with different doses of pyriproxyfen (water vs acetone: MD = 1.96; P = 0.11 and pyriproxyfen 23 ng vs pyriproxyfen 57 ng: MD = 1.99; P = 0.12; Fig 3). Conversely, both pyriproxyfen groups exhibited chemical profiles that were significantly different from the two control groups (water vs pyriproxyfen 23 ng: MD = 4.28; P < 0.001, water vs pyriproxyfen 57 ng: MD = 4.74; P < 0.001 and acetone vs pyriproxyfen 23 ng: MD = 3.93; *P* < 0.005, acetone vs pyriproxyfen 57 ng: MD = 5.32; P < 0.001).

**Table 2:** Percentage of correct assignments of pyriproxyfen-treated and control bees based on their cuticular hydrocarbon profiles.

Treatment	% correct assignment	Control water	Control acetone	Pyriproxyfen 23 ng	Pyriproxyfen 57 ng
Control water	71.4	15	4	2	0
Control acetone	71.4	2	15	3	1
Pyriproxyfen 23 ng	80	1	0	16	3
Pyriproxyfen 57 ng	75	0	1	4	15

#### Behavioral analyses on young honeybees (five day period after reintroduction with all control and treated bees)

**Fig 4:**
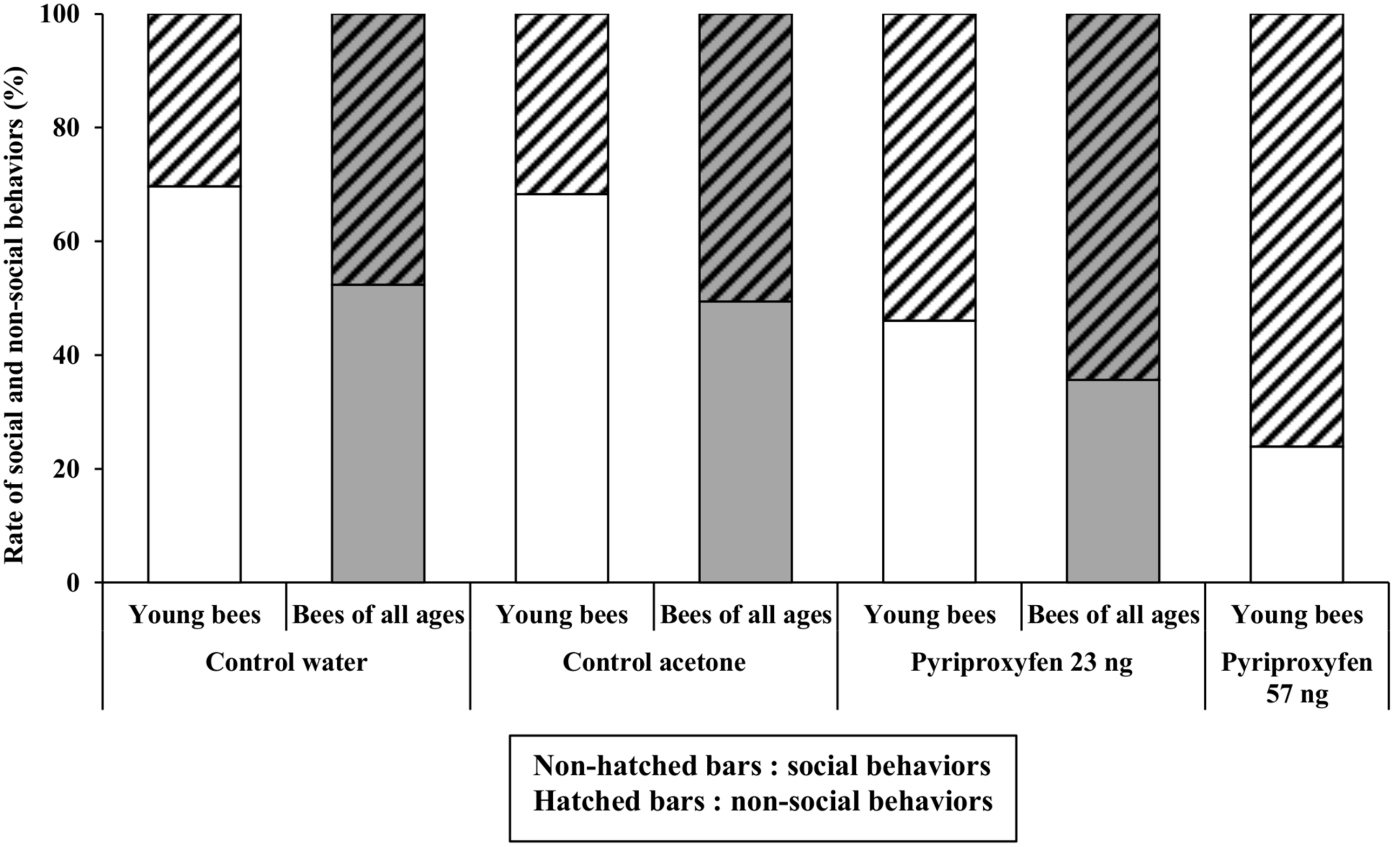
Percentage of social and non-social behavior occurrence in young honeybees (period of five days after reintroduction) for control and pyriproxyfen-treated bees, and in honeybees of all ages (overall period) for control bees and bees treated with 23 ng of pyriproxyfen. In young honeybees, percentages were established on 89 and 82 behaviors for control water and acetone bees, respectively, and 76 and 46 behaviors for pyriproxyfen 23 and 57 ng treated bees, respectively. In honeybees of all ages, percentages were established on 296 and 257 behaviors for control water and acetone bees, respectively and 174 behaviors for pyriproxyfen 23 ng treated bees.

GLMM analysis indicates that social behavior performance did not significantly differ between control groups (Tables 3 and 4). However, exposure to pyriproxyfen at 23 and 57 ng had a significant effect on social behavior performance compared to both control groups (Tables 3 and 4). Young pyriproxyfen-exposed bees performed more non-social tasks (self-grooming, inactivity, walking) compared to control bees in a dose-dependent manner (Fig 4). During the observation period, we did not find any age effect on social behavior performance.

**Table 3:** Statistical values of the generalized linear mixed-effect model using acetone as a control reference to determine the effect of treatments and age on social behavior occurrence in young bees (observations from day 3 to day 5 after reintroduction).

Covariate	Estimate ± S.E.	z value	*P*
**Intercept**	**2.578 ± 1.060**	**2.433**	**0.015**
Control water (n = 89)	-0.666 ± 1.471	-0.453	0.651
**Pyriproxyfen 23 ng (n = 76)**	**-5.173 ± 1.734**	**-2.984**	**0.003**
**Pyriproxyfen 57 ng (n = 46)**	**-6.805 ± 2.537**	**-2.682**	**0.007**
Age	-0.506 ± 0.283	-1.787	0.074
Control water x age	0.205 ± 0.394	0.521	0.602
**Pyriproxyfen 23 ng** x **age**	**1.143 ± 0.452**	**2.527**	**0.012**
Pyriproxyfen 57 ng x age	1.429 ± 0.730	1.958	0.050

GLMMs logistic regression was performed on a total of 293 behaviors. n indicates the number of behavioral observations per treatment group and *P* values refer to Wald Test probability.

**Table 4:** Statistical values of the generalized linear mixed-effect model using water as a control reference to determine the effect of treatments and age on social behavior occurrence in young bees (observations from day 3 to day 5 after reintroduction).

Covariate	Estimate ± S.E.	z value	*P*
**Intercept**	**1.912 ± 1.021**	**1.873**	**0.061**
Control acetone (n = 82)	0.666 ± 1.471	0.453	0.651
**Pyriproxyfen 23 ng (n = 76)**	**-4.507 ± 1.710**	**-2.636**	**0.008**
**Pyriproxyfen 57 ng (n = 46)**	**-6.139 ± 2.521**	**-2.435**	**0.015**
Age	-0.301 ± 0.273	-1.100	0.271
Control acetone x age	-0.205 ± 0.394	-0.521	0.602
**Pyriproxyfen 23 ng** x **age**	**0.938 ± 0.446**	**2.101**	**0.036**
Pyriproxyfen 57 ng x age	1.224 ± 0.726	1.686	0.092

GLMMs logistic regression was performed on a total of 293 behaviors. n indicates the number of behavioral observations per treatment group and *P* values refer to Wald Test probability.

#### Behavioral analyses throughout the total observation period (control and pyriproxyfen 23 ng treated bees)

For the overall period (15 days), GLMM analysis showed that the performance of social behavior did not significantly differ between bees from the two control groups (Tables 5 and 6). However, it varied significantly between control and pyriproxyfen 23 ng treated bees (Tables 5 and 6). Bees of all ages exposed to the JH analog expressed more non-social behaviors compared to control bees (Fig 4). When considering the whole observation period, the performance of social behavior was significantly affected by the age of the bees (Tables 5 and 6). We also found a significant 23 ng pyriproxyfen treatment by age interaction on social behavior compared to the control acetone by age interaction (Table 5). However there was no significant 23 ng pyriproxyfen treatment by age interaction compared to the water control by age interaction (Table 6).

**Table 5:** Statistical values of the generalized linear mixed-effect model using acetone as a control reference to determine the effect of treatments and age on social behavior occurrence in all bees (observations from day 3 to day 17 after reintroduction).

Covariate	Estimate ± S.E.	z value	*P*
**Intercept**	**1.421 ± 0.309**	**4.600**	**< 0.0001**
Control water (n = 296)	-0.239 ± 0.419	-0.571	0.568
**Pyriproxyfen 23 ng (n = 174)**	**-1.589 ± 0.479**	**-3.314**	**<0.001**
**Age**	**-0.184 ± 0.036**	**-5.103**	**< 0.0001**
Control water x age	0.055 ± 0.047	1.170	0.242
**Pyriproxyfen 23 ng** x **age**	**0.124 ± 0.060**	**2.070**	**0.038**

GLMMs logistic regression was performed on a total of 727 behaviors. n indicates the number of behavioral observations per treatment group and *P* values refer to Wald Test probability.

**Table 6:** Statistical values of the generalized linear mixed-effect model using water as a control reference to determine the effect of treatments and age on social behavior occurrence in all bees (observations from day 3 to day 17 after reintroduction).

Covariate	Estimate ± S.E.	z value	*P*
**Intercept**	**1.182 ± 0.283**	**4.183**	**< 0.0001**
Control acetone (n = 257)	0.239 ± 0.419	0.571	0.568
**Pyriproxyfen 23 ng (n = 174)**	**-1.349 ± 0.463**	**-2.916**	**0.004**
**Age**	**-0.129 ± 0.030**	**-4.264**	**< 0.0001**
Control acetone x age	-0.055 ± 0.047	-1.170	0.242
Pyriproxyfen 23 ng x age	0.069 ± 0.056	1.216	0.224

GLMMs logistic regression was performed on a total of 727 behaviors. n indicates the number of behavioral observations per treatment group and *P* values refer to Wald Test probability.

## Discussion

Due to the key role of JH in honeybee biology, it was reasonable to assume that JH analog-based insecticides, even at low doses, might affect the development and/or behaviors of exposed individuals. Our results confirmed this hypothesis and showed that sublethal doses of a JH analog can impact the social integration of bees in their colony (high rejection rate and low performance of social tasks). Exposure at the larval stage did not induce striking effects during development, but visible consequences did appear at the adult stage.

Previous studies conducted with JH analogs like pyriproxyfen mainly focused on developmental and/or physiological effects in bees [33–35]. High exposure levels were often tested in a range that could induce mortality and abnormalities, especially during development. Our cumulative tested doses of 23 and 57 ng of pyriproxyfen did not lead to additional mortality or abnormalities during development and were thus sublethal. In addition, despite the continuous exposure to pyriproxyfen, individuals successfully pass from the larval to the pupal stage (transition normally mediated by the decline of JH [36]); which suggests either a rapid degradation of pyriproxyfen or that this molecule has no effect on bee development at the tested doses. However, our treatment induced higher wing malformations in adults, which was already reported by Yang et al. [37] with concentrations of pyriproxyfen (0.1 and 1 ppm) relatively similar to ours. Developmental perturbation following pyriproxyfen exposure has also been shown at the muscular level, with a delay in flight muscle differentiation [38]. Comparison with field-relevant levels is difficult as few studies attempt to determine pyriproxyfen residues in plant nectar or pollen, beehive matrices or water in the field. Honeybee larvae can be exposed orally and by contact through brood food given by worker nurses (jelly), pollen and wax. It is still not possible to predict the dose received by larvae from brood food or wax but the dose received through resources like pollen can be predicted [39]. A recent study by Lambert et al. [40] reported mean concentrations of pyriproxyfen residue of 0.83 ppb in honey (limit of detection = 1.5 ppb) and 5.85 ppb in pollen (limit of detection = 2.1 ppb) from respectively 3.5 and 4.7% honeybee colonies in western France. According to Babendreier et al. [39] a larva receives 5.4 mg of pollen between the 3^rd^ and the 5^th^ day of its development. Moreover, the sugar content that larva receives from nurse food was estimated to be 59.4 mg with 5.4 mg within the first 3 days and 54 mg within the last 2 days [41]. If we consider the quantity of pollen consumed per larva and if we assume that sugar content received per larva is equal to nectar, then, based on Lambert et al. [40] data, a larva would only be exposed to 0.042 ng of pyriproxyfen during its development. Hence, our tested doses of 23 and 57 ng per larva are higher than this residual level. But, as we cannot really take into account exposure through brood food or through wax, exposure is underestimated. Authors like Zhu et al. [42] consider that applying 10 times the residual contents could give a realistic estimation of the actual exposure of larval bees through contaminated diet or direct transfer from residues in the comb. Pyriproxyfen residues were detected in beehive waxes in the United States, with 1% of samples containing a median content of 4.9 ppb (limit of detection = 1 ppb; [43]).

Larval pyriproxyfen exposure resulted in lower social task performance in adults. A physiological study investigating the effects of a larval JH application on adult behavioral development did not show any change in foraging age [44]. Authors hypothesized that JH treatments could have organizational effects on other aspects of foraging, or other behaviors. They also suggested that a JH analog could affect the development of brain structures, which would imply effects on workers’ cognitive abilities and social behavior performance, like brood care. Moreover, JH or JH analog treatments were reported to inhibit hypopharyngeal glands development involved in the production of royal jelly by worker bees to feed larvae [45,46]. Similarly, pyriproxyfen has been shown to affect vitellogenin synthesis in the hemolymph of workers [34]. This glycolipoprotein acts in concert with JH in the regulation of adult division of labor [47] and also possibly in the production of royal jelly [48]. These results were found after adult exposure, but an additional experiment of ours showed that repeated larval exposure to the highest expected dose of 54 ng per larva of pyriproxyfen induced significant negative effect on hypopharyngeal gland development of resulting adults (S2 Table). It has also been shown that administration of JH treatment or JH analogs at early developmental stages shortens development time in workers and results in queen-like features [33,44]. Indeed, JH appears to play a role in honeybee queen-worker caste determination during the larval stage [49,50], with queen-destined bees having a higher JH titer than worker-destined bees. Our results support the hypothesis of intercaste features with a more queen-like development time induced by early larval JH analog exposure to the two tested doses of pyriproxyfen (i.e. shorter development). Because of a more queen-like development in treated bees, we visually compared the morphology of adult control and treated bees but did not find any differences. Altogether, those results suggest that pyriproxyfen-treated bees would not be able to ensure their social tasks, like brood care, because they would not be physiologically adapted (acquisition of queen-worker intermediary features) or because they would have altered cognitive abilities. At the highest dose of exposure, hypopharyngeal gland development was also negatively affected (S2 Table), which supports the hypothesis that pyriproxyfen-exposed individuals are not physiologically adapted for brood care.

JH analog exposure induces egg or larval removal from brood cells by workers [12,51]. In our study, larval rearing was performed *in vitro*, removing the possible influence of nestmates on brood removal. Removal of treated larvae by workers could occur because of a change in the characteristics of larvae that nurses would recognize (e.g. behavior [52] or chemical profiles [27,53]). Here, we observed that larval pyriproxyfen treatment induced a higher and dose-dependent rejection rate of reintroduced adults by nestmates. At the same time, we found that pyriproxyfen exposure during the larval stage resulted in different CHC profiles in adults. This is interesting because CHC profiles are known to play an important role in nestmate recognition [26] and are very sensitive to the genotype of individuals [54], health status [28,55] and the environment [56,57]. For example, it has been shown that immune stimulation can change the CHC profiles of bees and lead to modified and aggressive interactions from nestmates [58,59]. Infection with the deformed wing virus also induced a modification of chemical profiles and a higher rate of bee rejection from the colony as compared to healthy individuals [60]. Therefore, one plausible explanation is that pyriproxyfen induces physiological changes that are reflected by CHC and at the origin of the distinction and rejection by nestmates. We also cannot exclude a possible age-related effect on CHC profiles [26] as pyriproxyfen-treated bees were of a younger age than controls (especially at the 57 ng dose) when rejected and thus analyzed (age range: 1 to 17 days old for water control bees; 2 to 17 days old for acetone control bees; 1 to 14 days old for pyriproxyfen 23 ng treated bees; 1 to 9 days old for pyriproxyfen 57 ng treated bees). However, despite a great overlap in the age of bees (e.g. controls vs pyriproxyfen 57 ng), the analysis of the corresponding CHC profiles revealed almost no overlap (Fig 3). It is also possible that intercaste features induced by pyriproxyfen played a role in the rejection of pyriproxyfen-treated bees. In the same manner, the low rejection rate observed for control bees could be due to in-vitro larval rearing conditions and especially the artificial diet containing 50% royal jelly which has the potential to confer some intercaste features [61].

A decrease in young honeybee renewal due to rejection by nestmates and/or because of workers that do not fulfill social tasks, like brood care, could have direct negative effects on population growth and balance of the colony. For instance, it has been stated that a decrease in brood and the number of emerging bees may be more damaging to the colony than the loss of foragers because the flexibility of division of labor and forager replacement rely heavily on sufficient brood and nurse bees (for a review see Thompson, [62]). However, when modeling the long-term effects of the JH analog fenoxycarb on the honeybee colony, Thompson et al. [13] showed that precocious foraging may have more impact than brood reduction. From the results of this study, Devillers et al. [63] modeled the long-term effects of pyriproxyfen and showed that a stress combining young bee removal and nurses with affected social task behavior may have deeper consequences for the colony than either stress alone. The intensity of the effects on colonies would also depend on the stress period [13,61,64]. When simulating stress at different dates, Devillers et al. [63] showed that an early spring application in March, when the colony starts its development, or later in the season in July or August, when winter bees are produced, can be more critical for the colony fate than stress at other dates.

In the current context of climate change, we can expect to witness an increased use of biocides and the development of new biocides to control the expansion of mosquito populations and vector-borne disease reemergence. This raises the question about their potential impacts on beneficial insects like honeybees. Honeybee foragers frequently visit aquatic areas where mosquitoes breed to collect water necessary for the colony, especially for brood rearing [65]. Thus, when applied as a larvicide for vector control in wet areas, biocide residues, such as the JH analog pyriproxyfen, would need to be estimated.

## Supporting Information

S1 Table Relative proportion of each cuticular hydrocarbon in control and pyriproxyfen-treated bees. (DOCX)

S2 Table Protein content (mg) in the heads of control and pyriproxyfen treated-bees. (DOCX)
