# Supplementary material for: Larval Exposure to the Juvenile Hormone Analog Pyriproxyfen Disrupts Acceptance of and Social Behavior Performance in Adult Honeybees
Source: PLoS One. 2015 Jul 14;10(7):e0132985. doi: 10.1371/journal.pone.0132985 (PMC4501706; doi:10.1371/journal.pone.0132985)
Supplement: S1 Table — (DOCX) [file pone.0132985.s001.docx]

**Table S1. Relative proportion of each cuticular hydrocarbon in control and pyriproxyfen-treated bees.** Changes induced by the treatments were determined with Kruskal-Wallis tests.

|  | **Control**  **water** | **Control acetone** | **Pyriproxyfen**  **23 ng** | **Pyriproxyfen 57 ng** | **p-value** |
| --- | --- | --- | --- | --- | --- |
| **Heneicosane** | 0.89+0.11 | 0.96+0.09 | 1.19+0.08 | 1.24+0.11 | 0.01 |
| **Tricosane** | 5.35+0.58 | 5.04+0.36 | 7.32+0.89 | 6.03+0.38 | 0.03 |
| **Pentacosane** | 7.24+0.60 | 7.40+0.48 | 9.63+0.81 | 9.41+0.53 | <0.001 |
| **Heptacosane** | 17.3+1.07 | 18.8+1.02 | 21.9+1.11 | 24.9+1.09 | < 0.001 |
| **Nonacosane** | 14.7+0.68 | 15.9+0.56 | 13.1+0.54 | 13.3+0.57 | <0.01 |
| **Triacontane** | 0.43+0.02 | 0.46+0.03 | 0.39+0.02 | 0.41+0.02 | 0.41 |
| **Hentriacontane** | 9.95+0.54 | 10.1+0.57 | 9.07+0.57 | 9.01+0.70 | 0.34 |
| **Dotriacontane** | 2.46+0.35 | 2.78+0.50 | 2.87+0.49 | 2.90+0.52 | 0.78 |
| **Tritriacontane** | 1.39+0.11 | 1.40+0.12 | 1.39+0.11 | 1.30+0.15 | 0.69 |
| **Tricosene Isomère 1** | 4.68+0.87 | 5.35+0.97 | 2.52+0.36 | 0.98+0.24 | < 0.001 |
| **Tricosene Isomère 2** | 0.58+0.11 | 0.56+0.04 | 0.60+0.05 | 0.61+0.06 | 0.54 |
| **Pentacosene** | 0.83+0.13 | 0.70+0.04 | 0.71+0.04 | 0.80+0.08 | 0.92 |
| **Heptacosene** | 0.54+0.06 | 0.53+0.03 | 0.54+0.02 | 0.74+0.10 | 0.29 |
| **Hentriacontene Isomère 1** | 7.20+0.53 | 6.82+0.47 | 5.18+0.24 | 4.59+0.28 | < 0.001 |
| **Hentriacontene isomère 2** | 5.24+0.34 | 4.73+0.21 | 4.11+0.18 | 3.70+0.22 | < 0.001 |
| **Tritriacontene** | 12.5+1.00 | 10.0+0.59 | 11.4+0.62 | 10.7+0.72 | 0.20 |
| **Tritriacont-diène** | 3.41+0.36 | 2.91+0.31 | 2.14+0.11 | 2.20+0.14 | 0.029 |
| **11,13-Méthylheptacosane** | 2.00+0.32 | 2.19+0.25 | 2.49+0.33 | 2.85+0.17 | <0.01 |
| **11, 13,15-Méthylnonacosane** | 1.65+0.33 | 1.57+0.15 | 1.84+0.22 | 2.33+0.12 | < 0.001 |
| **11, 13,15-Méthylhentriacontane** | 0.82+0.17 | 0.75+0.07 | 0.90+0.10 | 1.16+0.07 | < 0.001 |
| **11,13,15,17 -Méthyltritriacontane** | 0.66+0.11 | 0.63+0.12 | 0.54+0.08 | 0.51+0.07 | 0.68 |
