# Supplementary material for: Larval Exposure to the Juvenile Hormone Analog Pyriproxyfen Disrupts Acceptance of and Social Behavior Performance in Adult Honeybees
Source: PLoS One. 2015 Jul 14;10(7):e0132985. doi: 10.1371/journal.pone.0132985 (PMC4501706; doi:10.1371/journal.pone.0132985)
Supplement: S2 Table — (DOCX) [file pone.0132985.s002.docx]

**Table S2. Protein content (mg) in the heads of control and pyriproxyfen treated-bees.** Protein content, used to assess the development of hypopharyngeal glands, was measured in 8 days-old bees with the Bradford method*. Two trials were performed and mean ± SD and number of bees (n) are shown. One-way ANOVA (P < 0.05) followed by Dunnett tests (P < 0.05) were performed to compare protein content between treatments. Different letters indicate significant differences (P < 0.05).

|  | **Control water** | **Control acetone** | **Pyriproxyfen 18 ng** | **Pyriproxyfen 54 ng** |
| --- | --- | --- | --- | --- |
| **Trial 1** | 0.33 ± 0.06^a^  (n= 30) | 0.34 ± 0.09^a^  (n=19) | 0.35 ± 0.08^a^  (n=24) | 0.25 ± 0.03^b^  (n=17) |
| **Trial 2** | 0.38 ± 0.11^a^  (n= 25) | 0.35 ± 0.15^a^  (n=18) | 0.33 ± 0.06^a^  (n=16) | 0.25 ± 0.06^b^  (n=23) |

Nominal doses of pyriproxyfen are given.

*Fortini D, Michaud B, Aupinel P. Comparison of two methods to assess effects of insecticides on hypopharyngeal gland development of honey bee. Hazards of pesticides to bees – 10th International Symposium of the ICP-Bee Protection Group. 2009;423:102.
